# Editorial: Cell cross-talk in diabetic kidney diseases, volume III

**DOI:** 10.3389/fmed.2025.1627354

**Published:** 2025-05-30

**Authors:** Ruopei Wang, Lu Zhang

**Affiliations:** Department of Nephrology (Fujian Provincial Clinical Research Center for Glomerular Nephritis), The First Affiliated Hospital of Xiamen University, School of Medicine, Xiamen University, Xiamen, China

**Keywords:** diabetic kidney disease, cell cross-talk, chronic kidney disease, glomerular endothelial cells, podocytes

Diabetic kidney disease (DKD) has emerged as a major public health challenge of the 21st century, with approximately one-third of diabetic patients worldwide developing DKD. This condition is a leading cause of end-stage renal disease and cardiovascular complications, placing a significant burden on healthcare systems (1).

Despite the advent and implementation of comprehensive therapeutic strategies for DKD—including a four-cornerstone combination of renin-angiotensin-aldosterone system inhibitors (RAASi), sodium-glucose cotransporter-2 inhibitors (SGLT2i), mineralocorticoid receptor antagonists (MRA), and glucagon-like peptide-1 receptor agonists (GLP-1 RA) (2, 3), —the progression of DKD remains a formidable clinical challenge. Current interventions, though beneficial, provide only partial efficacy in halting disease progression, underscoring the urgent need for novel and more effective treatment modalities.

A comprehensive understanding of the complex pathogenesis of DKD, the development of innovative therapeutic strategies, and the implementation of effective preventive measures are therefore of paramount importance (4). In recent years, research has increasingly focused not on the mechanisms of individual cell types, but on the interactions among different cells, as highlighted in this topic series. We have successfully published two Research Topic that have effectively presented research outcomes related to this field. This editorial includes four papers that synthesize the key findings of recent studies, highlighting their contributions to our understanding of DKD and their implications for future research and treatment strategies.

Zhuang et al. investigated the prevalence of chronic kidney disease (CKD) from an epidemiological perspective, providing valuable insights into the burden of DKD. Their analysis revealed that the rising prevalence of diabetes significantly contributed to the increased incidence of early-stage CKD in the United States between 1999 and 2018. These findings highlight the urgent need to elucidate the pathogenesis of DKD at the cellular level. Furthermore, the identification of key risk factors—such as age, diabetes status, and racial disparities—offers a critical framework for examining how these variables may influence intercellular communication in DKD. While epidemiological data underscore the extensive impact of DKD, a deeper understanding of its molecular mechanisms remains essential for the development of targeted and effective therapeutic strategies.

Chatterjee et al. provided a comprehensive overview of cellular cross-talk in DKD, emphasizing its pivotal role in disease pathogenesis. Their work highlights that intercellular communication extends beyond glomerular resident cells—podocytes (PCs), mesangial cells (MCs), and glomerular endothelial cells (GECs)—to include the tubulo-glomerular axis, immune cells, and components of the innate immune system. In the early stages of DKD under high-glucose (HG) conditions, NADPH oxidase is activated in GECs, resulting in increased production of reactive oxygen species (ROS), which in turn activates PCs. Moreover, HG-induced, GEC-derived exosomes enhance the expression of fibronectin and collagen IV genes in MCs. Simultaneously, MCs secrete Semaphorin 3C (SEMA3C), which disrupts glomerular permeability. Thus, GEC injury can lead to podocyte damage, while podocyte loss further exacerbates GEC dysfunction, establishing a self-reinforcing pathological cycle.

These interconnected events collectively contribute to mesangial expansion—a hallmark pathological feature of DKD. This intricate cellular interplay underscores the complexity of DKD progression and highlights potential therapeutic targets.

With a clearer understanding of DKD pathogenesis, research attention has increasingly focused on the development of effective therapeutic strategies. In this context, Fan et al. provide novel insights into the role of exercise as an adjunctive treatment for DKD. Their findings suggest that exercise modulates key cellular and molecular pathways—including mTORC1, CaMKII, AMPK, and IRS1/PI3K/AKT/GLUT4—thereby improving glucose homeostasis, enhancing insulin sensitivity, and promoting glucose uptake, all of which are critical for mitigating DKD progression.

Beyond glycemic control, exercise exerts renoprotective effects by regulating the RAAS, reducing renal oxidative stress and inflammation, improving endothelial function, modulating lipid metabolism, and stimulating mitochondrial biogenesis. Notably, exercise also influences the muscle–kidney axis. During physical activity, skeletal muscles release myokines; for example, sustained exercise lowers interleukin-6 (IL-6) levels and systemic inflammation. Certain myokines, such as irisin, can inhibit transforming growth factor-beta 1 (TGF-β1) signaling and activate AMPK, thereby reducing renal fibrosis and protecting renal function. However, other myokines, such as lactate, may exacerbate oxidative stress, and their effects may vary depending on exercise intensity—a subject warranting further investigation. Collectively, these findings position exercise as a promising, low-risk, and cost-effective adjunct therapy for patients with DKD, offering benefits that extend beyond those of conventional pharmacological interventions.

Although treatment is essential for the management of DKD, prevention is regarded as the most effective long-term strategy. The research by Wang et al. offers valuable insights into the prevention of DKD in patients with type 2 diabetes mellitus (T2DM). Their findings indicate that vitamin D, a fat-soluble micronutrient, exerts renoprotective effects. Vitamin D may act through multiple cellular mechanisms. For instance, it can regulate the RAAS, improve insulin sensitivity, reduce inflammation, delay the onset of proteinuria, and enhance autophagy in renal cells. Moreover, deficiencies in vitamins B and K have also been implicated in the pathogenesis of DKD (5, 6). This underscores the importance of regularly monitoring vitamin levels in patients at risk for DKD and providing appropriate vitamin supplementation, which may serve as an effective preventive strategy to reduce the incidence and progression of DKD.

The research findings presented in these four articles collectively highlight the importance of further investigating cellular cross-talk in DKD. Furthermore, the development of targeted therapeutic strategies based on these elucidated mechanisms is of critical importance. Therapies that can modulate the activity of specific signaling pathways involved in cellular cross-talk, such as the TGF-β pathway (7), have the potential to offer novel treatment options for patients with DKD (8–12). In addition, well-designed clinical trials should be conducted to evaluate the combined effects of these interventions on DKD progression, while accounting for individual patient characteristics such as age, ethnicity, and comorbid conditions. Moreover, the use of single-cell RNA sequencing (scRNA-seq) is highly recommended for studying cellular cross-talk (13). Such scRNA-seq based analyses can provide high-resolution insights into cellular heterogeneity and gene regulatory mechanisms within complex biological systems. Furthermore, mechanisms identified through scRNA-seq studies can be further validated in clinical cohorts using serum and urine mass spectrometry analyses (3).

In summary, research on cellular cross-talk in DKD has yielded invaluable insights into the pathogenesis of this condition. A comprehensive understanding of these complex cellular interactions will facilitate the development of more effective therapeutic strategies and ultimately improve the prognosis of patients with DKD. Future research should continue to investigate these mechanisms and focus on translating findings into practical clinical applications, thereby addressing the growing global burden of DKD.
